# Differentiation of benign and malignant lymph nodes in pediatric patients on ferumoxytol-enhanced PET/MRI

**DOI:** 10.7150/thno.40606

**Published:** 2020-02-18

**Authors:** Anne Monika Muehe, Florian Siedek, Ashok Joseph Theruvath, Jayne Seekins, Sheri L. Spunt, Allison Pribnow, Florette Kimberly Hazard, Tie Liang, Heike Daldrup-Link

**Affiliations:** 1 Department of Radiology, Molecular Imaging Program at Stanford, Stanford University, Stanford, CA, USA; 2 Institute of Diagnostic and Interventional Radiology, University of Cologne, Faculty of Medicine and University Hospital Cologne, Cologne, Germany; 3 Department of Diagnostic and Interventional Radiology, University Medical Center of the Johannes Gutenberg-University Mainz, Mainz, Germany; 4 Department of Pediatrics, Division of Hematology and Oncology, Lucile Packard Children's Hospital, Stanford University, Stanford, CA, USA; 5 Department of Pathology, Division of Hematology and Oncology, Lucile Packard Children's Hospital, Stanford University, Stanford, CA, USA

**Keywords:** nanoparticles, PET/MRI, lymph nodes, cancer imaging, ferumoxytol

## Abstract

The composition of lymph nodes in pediatric patients is different from that in adults. Most notably, normal lymph nodes in children contain less macrophages. Therefore, previously described biodistributions of iron oxide nanoparticles in benign and malignant lymph nodes of adult patients may not apply to children. The purpose of our study was to evaluate if the iron supplement ferumoxytol improves the differentiation of benign and malignant lymph nodes in pediatric cancer patients on ^18^F-FDG PET/MRI.

**Methods:** We conducted a prospective clinical trial from May 2015 to December 2018 to investigate the value of ferumoxytol nanoparticles for staging of children with cancer with ^18^F-FDG PET/MRI. Ferumoxytol is an FDA-approved iron supplement for the treatment of anemia and has been used “off-label” as an MRI contrast agent in this study. Forty-two children (7-18 years, 29 male, 13 female) received a ^18^F-FDG PET/MRI at 2 (n=20) or 24 hours (h) (n=22) after intravenous injection of ferumoxytol (dose 5 mg Fe/kg). The morphology of benign and malignant lymph nodes on ferumoxytol-enhanced T2-FSE sequences at 2 and 24 h were compared using a linear regression analysis. In addition, ADCmean-values, SUV-ratio (SUV_max_ lesion/SUV_mean_ liver) and R2*-relaxation rate of benign and malignant lymph nodes were compared with a Mann-Whitney-U test. The accuracy of different criteria was assessed with a receiver operating characteristics (ROC) curve. Follow-up imaging for at least 6 months served as the standard of reference.

**Results:** We examined a total of 613 lymph nodes, of which 464 (75.7%) were benign and 149 (24.3%) were malignant. On ferumoxytol-enhanced T2-FSE images, benign lymph nodes showed a hypointense hilum and hyperintense parenchyma, while malignant lymph nodes showed no discernible hilum. This pattern was not significantly different at 2 h and 24 h postcontrast (p=0.82). Benign and malignant lymph nodes showed significantly different ferumoxytol enhancement patterns, ADCmean values of 1578 and 852 x10^-6^ mm^2^/s, mean SUV-ratios of 0.5 and 2.8, and mean R2*-relaxation rate of 127.8 and 84.4 Hertz (Hz), respectively (all p<0.001). The accuracy of ADCmean, SUV-ratio and pattern (area under the curve (AUC): 0.99; 0.98; 0.97, respectively) was not significantly different (p=0.07). Compared to these three parameters, the accuracy of R2* was significantly lower (AUC: 0.93; p=0.001).

**Conclusion:** Lymph nodes in children show different ferumoxytol-enhancement patterns on MRI than previously reported for adult patients. We found high accuracy (>90%) of ADCmean, SUV-ratio, pattern, and R2* measurements for the characterization of benign and malignant lymph nodes in children. Ferumoxytol nanoparticle accumulation at the hilum can be used to diagnose a benign lymph node. In the future, the delivery of clinically applicable nanoparticles to the hilum of benign lymph nodes could be harnessed to deliver theranostic drugs for immune cell priming.

## Introduction

Accurate detection of lymph node metastases is important for cancer staging of pediatric patients 1-3. In the clinical realm, the morphology of lymph nodes is commonly used to estimate malignancy: Lymph nodes with a size of more than 1 cm and round shape are considered malignant on ultrasound 4, computed tomography (CT) 5, 6 or magnetic resonance imaging (MRI) 7. Unsurprisingly, these morphological criteria demonstrate low specificity because malignant tumor cell infiltrations can be found in subcentimeter nodes and infections can cause enlarged benign nodes 6. More recently, an increased glucose metabolism, as measured by an increased uptake of ^18^F-fluorodeoxyglocose (^18^F-FDG) on a positron emission tomography (PET) scan, provided improved specificity for the detection of malignant lymph nodes in children with lymphoma 8, soft tissue sarcoma 9 and bone sarcoma 10, among others 11. However, ^18^F-FDG PET studies can still be difficult to interpret in children, because they often have inflammatory nodes 12: Previous investigators reported an incidence of 27% false positive benign lymph nodes on ^18^F-FDG PET studies in pediatric oncology patients 5, 13, 14 compared to 15% in adult patients 15. Therefore, additional approaches to diagnose malignant lymph nodes more reliably would be highly valuable 16.

Iron oxide nanoparticles can improve the specificity of MR imaging for the differentiation of benign and malignant lymph nodes 17, 18. In adult patients, benign lymph nodes showed significant T2- and T2*-enhancement of both hilum and parenchyma at 24 hours (h) after intravenous infusion of iron oxide nanoparticles, while malignant lymph nodes showed no significant enhancement 17-21. This difference was due to presumed nanoparticle phagocytosis by macrophages in benign lymph nodes and absent phagocytosis in metastatic lymph nodes, in which normal stroma cells were replaced by tumor cells 17.

However, this concept might not apply to pediatric patients due to well-described differences in the cellular composition of benign lymph nodes in children and adults 22, 23. In children, lymph nodes are better vascularized than in adults 24, the hilum contains more vessels and minimal fat 25 and the parenchyma is mostly composed of T- and B-cells 26, 27. Importantly, lymph nodes in children contain less macrophages 28. Therefore, the iron oxide nanoparticle enhancement patterns of benign lymph nodes in children could be different from those described for adult patients in the literature 17, 18. We hypothesized that the iron oxide nanoparticle enhancement of lymph nodes in children would be a function of vascularity rather than macrophage content. We investigated the iron oxide nanoparticle compound ferumoxytol, which is an FDA-approved iron supplement for the treatment of anemia, which can be used “off-label” as an MRI contrast agent. To our knowledge, the value of ferumoxytol nanoparticles for the differentiation of benign and malignant lymph nodes in pediatric patients has not been investigated. The purpose of our study was to evaluate if ferumoxytol administration improves the characterization of malignant lymph nodes in pediatric patients on ^18^F-FDG PET/MRI.

## Methods

### Patient population

This prospective, non-randomized, health insurance portability and accountability act-compliant clinical trial (NCT01542879) was approved by our institutional review board and performed under an investigator-initiated investigative new drug application (111,154). We included pediatric cancer patients from May 2015 until December 2018 if they met the following inclusion criteria: 1) age 6 to 18 years, 2) solid extracranial tumor, diagnosed based on standard imaging tests or biopsy, 3) willingness to give written informed consent. Patients were excluded if they had: 1) a primary lymphatic disease (i.e. lymphoma), 2) contraindications to MRI, 3) hemosiderosis or hemochromatosis, or 4) if they were pregnant. All patients or their legal representative gave written informed consent. We recruited 42 patients with biopsy proven sarcoma (osteosarcoma (n=11), Ewing sarcoma (n=4), soft tissue sarcoma including rhabdomyosarcoma (n=3), synovial sarcoma (n=3), undifferentiated sarcoma (n=2), gastrointestinal stromal tumor (n=1), desmoplastic small round cell tumor (n=3), malignant peripheral nerve sheath tumor (n=1)) as well as Langerhans cell histiocytosis (n=6), Wilms tumor (n=2), nasopharynxgeal carcinoma, ovarian steroid cell tumor, hepatocellular carcinoma, ovarian mixed germ cell tumor, neuroblastoma and lipoma (n=1, respectively). The patients included 13 girls (mean age 13.4 +/- 3.4 years; range: 7-18 years) and 29 boys (mean age 13.6 +/- 3.1 years; range: 8-18 years, Table S1).

### PET/MR-imaging

All patients underwent an integrated ^18^F-FDG PET/MRI scan (Signa PET/MR, GE Healthcare, Milwaukee, WI) at either 2 h (n=20) or 24 h (n=22) after an intravenous infusion of the iron supplement ferumoxytol (Feraheme® Injection, AMAG Pharmaceuticals, Waltham, MA). We randomly assigned patients to scans at two time points, 2 h and 24 h postcontrast, because previous studies had used delayed 24 hour scans for lymph node imaging 17, 19, 29. As a sub-aim of our investigations, we wanted to evaluate if the same information could be obtained from an earlier postcontrast scan, which would be more practical in a clinical-translational setting.

Ferumoxytol was diluted 1:3 in saline and administered at a dose of 5 mg Fe/kg bodyweight over 15 minutes (min) as recommended by the food and drug administration (FDA) 3. We carefully monitored the heart rate and blood pressure of the patients before, during and up to 30 min after the ferumoxytol infusion and after the patient finished the PET/MRI scan. We also asked the patients for any subjective side effects. At approximately 60 min before the planned PET/MRI scan (mean: 57.5 +/- 10.1 min, range: 42-86 min), all patients received an intravenous injection of ^18^F-FDG at a dose of 3 MBq/kg bodyweight (151.7 +/- 51.8 MBq, range: 62.9-270.1 MBq). After fasting for at least 4 h before the scan, the mean blood glucose level of all patients was 90.9 +/- 9.7 mg/dL (range: 71-109 mg/dL) at the time of the ^18^F-FDG injection. The effective dose from the ^18^F-FDG injection was calculated using the age-specific conversion factors published by the international commission on radiological protection 30.

Our imaging protocol included axial PET acquisition slabs (25 cm axial FOV, acquisition time of 3:30 min per slab) with simultaneous acquisition of the following axial MR sequences: T1-weighted Liver Acquisition with Volume Acquisition (LAVA), T2-weighted Fast Spin Echo (FSE) with fat saturation and diffusion-weighted imaging (DWI; b-value: 50/600). In addition, axial free-breathing periodically rotated overlapping parallel lines with enhanced reconstruction (PROPELLER) images of the chest and axial iterative decomposition of water and fat with echo asymmetry and least-squares estimation (IDEAL-IQ) images were obtained to create R2* maps. Detailed sequence parameters for PET/MRI scans are given in Table S2.

We reconstructed PET data using a 3D time of flight iterative ordered subsets expectation maximization algorithm (24 subsets, 3 iterations, temporal resolution 400 ps, matrix 192 × 192; voxel size 2.8×2.8×2.8 mm). A two-point Dixon MR sequence was used for attenuation correction.

### Morphology of benign and malignant lymph nodes

To evaluate morphological characteristics of benign and malignant lymph nodes, two radiologists (AJT, FS) measured the size (short and long axis) of all visually detectable lymph nodes on T2-weighted FSE images in five anatomical regions (neck, axilla, mediastinum, abdomen, and groin) of each patient. Response evaluation criteria for solid tumors (RECIST) guidelines were used to categorize benign and malignant lymph nodes based on their length of the short axis of <10 mm or ≥10 mm, respectively 31, 32. In order to determine if ferumoxytol enhancement can add diagnostic information, two radiologists (HD, AJT; >20 years and 7 years of experience) jointly reviewed and determined qualitative enhancement patterns for all lymph nodes. Twenty-seven of our patients had received a clinical scan with unenhanced axial T2-weighted FSE sequences (same sequence parameter as PET/ MRI-protocol) within three weeks (range: 1-21 days) before the PET/MRI scan. These unenhanced scans served as controls to determine the enhancement signal of the lymph nodes after ferumoxytol administration in children.

### Quantitative evaluation of benign and malignant lymph nodes

Two operators (FS and AJT) outlined each lymph node with operator defined regions of interest (ROIs) and measured the following parameters: ADCmean, R2* and SUV-ratio:

We reconstructed apparent diffusion coefficient (ADC) maps from DWI images (b=50/600) using the integrated OsiriX (OsiriX 8.2, Pixmeo, Geneva, Switzerland) software tool and measured the mean ADC value of each lymph node through an operator-defined region of interest (ROI), which covered the entire lymph node (i.e. the hilum and the parenchyma):

ADC_mean_ = 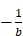 ln 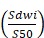


We generated R2* maps from multi-echo IDEAL-IQ sequences with the PET/MRI scanner software DV26. We measured the R2* relaxation rate of each lymph node using OsiriX software through operator-defined ROIs. To quantify the uptake of ferumoxytol nanoparticles in lymph nodes, we calculated R2* relaxation rates as R2*=1/T2*. R2* relaxation rates are directly proportional to the tissue's iron content 33.

We measured the ^18^F-FDG radiotracer uptake in lymph nodes and liver on the attenuation corrected PET data and calculated the maximum standardized uptake value (SUV_max_) of each lymph node with MIM software (MIM 6.5, Cleveland, OH).

SUV_max_ =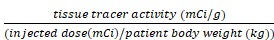


We normalized the SUV_max_ of each lymph node by the SUV_mean_ of the patient's liver as an internal standard of reference by calculating the SUV tumor-to-liver ratio. This allowed us to compare the ^18^F-FDG uptake in lymph nodes of different patients, who might have received their PET scans at slightly different time points after the ^18^F-FDG injection. A SUV-ratio above one indicates an increased tissue ^18^F-FDG uptake in lymph nodes compared to liver as an internal reference standard.

### Standard of reference

Follow up imaging for at least six months served as the standard of reference. A lymph node with benign imaging features that remained stable at 6 months was defined as benign. A lymph node with malignant features that either progressed or responded after chemotherapy was defined as malignant.

We obtained exemplary proof-of-concept histopathologies of a benign and a malignant lymph node in two of our patients, who had undergone a tumor resection along with a resection of lymph nodes one day after the ferumoxytol PET/MRI. The lymph node specimens were embedded in paraffin, sliced in 4-mm sections that were placed on glass slides. These slides were baked for 1 h at 60ºC and afterwards deparaffinized in xylene and hydrated in a graded series of alcohol. Hydrogen peroxide was used to block endogenous peroxidase. Samples were stained on the Benchmark XT stainer (Roche Ventana, Oro Valley, AZ) with standard heat-induced epitope retrieval except as indicated, and diaminobenzidine (Enzo Biochem Inc., Farmingdale, NY) as the chromogen. CD68 (mouse monoclonal KP1 1:1600, Dako, Santa Clara, CA) was the antibody used for macrophage staining. Hematoxylin (VWR, Radnor, PA) was used for counterstaining. To detect intracytoplasmatic iron in macrophages, paraffin-embedded lymph node slides were stained with Prussian blue (Dako, Santa Clara, CA). Light microscopic images were captured on an Olympus BX45 microscope (Olympus, Center Valley, PA) with UPlanFL 4 /0.13, 20/0.50, and 100/1.25 oil immersion lenses and a SpotFLEX camera.

### Statistical Analysis

Differences in morphology between benign and malignant lymph nodes at 2 h and 24 h post ferumoxytol injection were compared with a linear regression analysis using a general linear model. ADCmean, SUV-ratio and R2* of benign and malignant lymph nodes were compared with a Mann-Whitney-U test. ADCmean, SUV-ratio and R2* as predictors were evaluated with size and p-value, 95% confidence interval (CI), odds ratio (OR), area under the curve (AUC), and 95%CI of AUC were reported and compared using a general linear model with a random effect for each patient. Due to multiple comparisons (n=12), the corrected criterion for significance testing used was p=0.004 (0.05 divided by 12).

## Results

The ferumoxytol infusion was well tolerated by all patients and we did not encounter any adverse events. The mean radiation exposure of our patients as part of the ^18^F-FDG PET/MRI study was 2.8 mSv (range 1.2 - 5.2 mSv).

### Size of benign and malignant lymph nodes on ferumoxytol-enhanced MR scans

We detected a total of 613 lymph nodes in 42 patients. Of those, 464 (75.7%) were benign and 149 (24.3%) were malignant (Table S3). The mean short axis diameter was 5.5 +/- 3.5 mm for benign lymph nodes and 10.6 +/- 3.9 mm for malignant lymph nodes. Size is usually a major parameter for radiologists to assess if a lymph node is malignant, with larger than 10 mm considered malignant 4-6 and smaller than 5 mm being benign 6, 34. Yet, our data revealed size to be an insufficient predictor of malignant lymph nodes smaller than 10 mm with a sensitivity of only 47.7%. However, size was an excellent predictor of benign etiology for lymph nodes smaller than 5 mm with a sensitivity of 94.0%. Thus, the state of malignancy of lymph nodes cannot be predicted accurately within the range of 5-10 mm. Therefore, additional imaging criteria like iron oxide nanoparticle enhancement pattern would be most useful for lymph nodes in this range of sizes.

### Morphology of benign and malignant lymph nodes on ferumoxytol-enhanced MR scans

**Figure 1:**
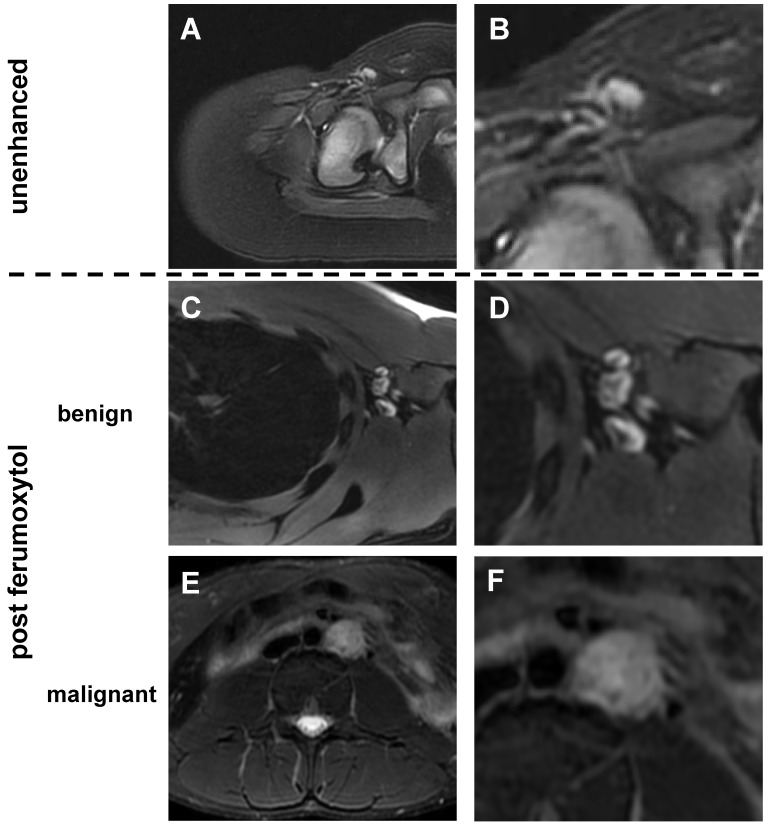
** Morphology of benign and malignant lymph nodes on T2-weighted FSE images**: (A) Normal lymph node in the right inguinal region demonstrates homogenous hyperintense signal before ferumoxytol administration. In young children, we found little or no fat at the lymph node hilum. (B) Enlarged view shows homogenous hyperintense lymph node with vessel entering the lymph node at the hilum. No significant fat is noted at the hilum. (C) After ferumoxytol administration, normal lymph node demonstrates dark (hypointense) enhancement of the hilum, but not the parenchyma. (D) Enlarged view demonstrates hypointense (dark) hilum and hyperintense (bright) parenchyma. (E) After ferumoxytol administration, malignant abdominal lymph node in the left para-aortic region demonstrates slightly inhomogeneous hyperintense T2-signal. (F) Enlarged view shows no evidence for hilar nanoparticle enhancement. FSE: fast spin echo.

**Figure 2:**
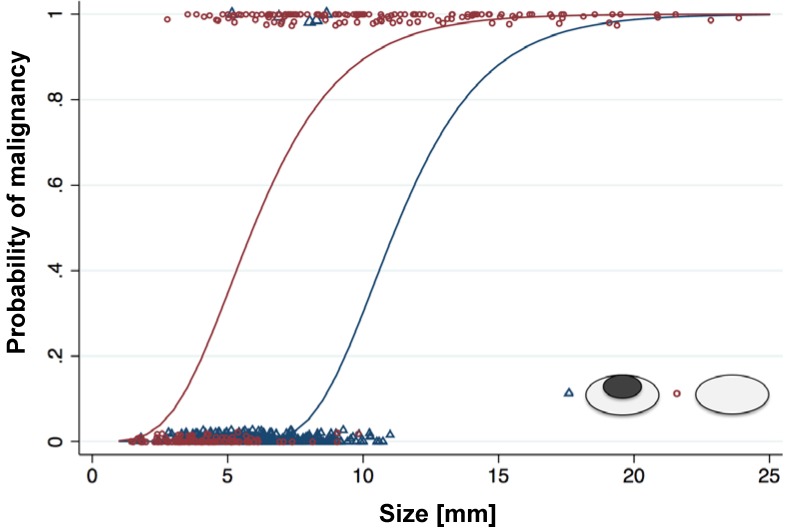
** Probability of malignancy of lymph nodes with different size and morphology on ferumoxytol-enhanced T2-FSE images:** For lymph nodes with a size of more than 10 mm, the probability of malignancy is high, and morphology on MRI adds little additional information. For lymph nodes with a size of less than 5 mm, the probability of malignancy is low, and morphology on MRI adds little additional information. For lymph nodes with a short axis diameter between 5-10 mm, morphology on ferumoxytol-enhanced MRI can help to differentiate benign and malignant nodes.

Unenhanced T2-FSE images showed a hyperintense signal of both benign and malignant lymph nodes: 289 (87%) of 333 benign lymph nodes did not show a fatty hilum and only 44 (13%) benign lymph nodes did show a fatty hilum. By contrast, T2-FSE images after ferumoxytol infusion delineated a hypointense hilum in 362 out of 464 benign lymph nodes (78.0%), whereas 144 out of 149 malignant lymph nodes were homogenously hyperintense (96.6 %) (Figure 1, Table 1). This difference in ferumoxytol- enhancement of benign and malignant lymph nodes was significant (p<0.001). The ferumoxytol enhancement pattern did not change significantly between 2 h and 24 h scans, neither for benign lymph nodes (p=0.01, NOTE: due to multiple testing significance level p=0.004) nor malignant lymph nodes (p=0.95, Figure S1). When considering nodal size, we found absent hilar enhancement in 79/79 (100%) malignant lymph nodes > 10 mm and positive hilar enhancement in 10/11 (91%) benign lymph nodes > 10 mm (Figure S2). For sizes ≥ 5 - <10 mm, pattern added significant value compared to size alone (p<0.001) with a ten-fold higher chance of malignancy for lymph nodes with absent hilar enhancement (OR size: 1.4, OR pattern: 10.9; Figure 2, Table 2). We did not recognize the hypointense hilum in 74 (39.8%) out of 186 benign lymph nodes < 5 mm (Figure S2). Furthermore, presence or absence of hilar enhancement yielded no additional information for nodes < 5 mm in size, because the likelihood of them being malignant was so low with a sensitivity of 94.0%.

### Quantitative evaluation of benign and malignant lymph nodes in pediatric patients

**Figure 3:**
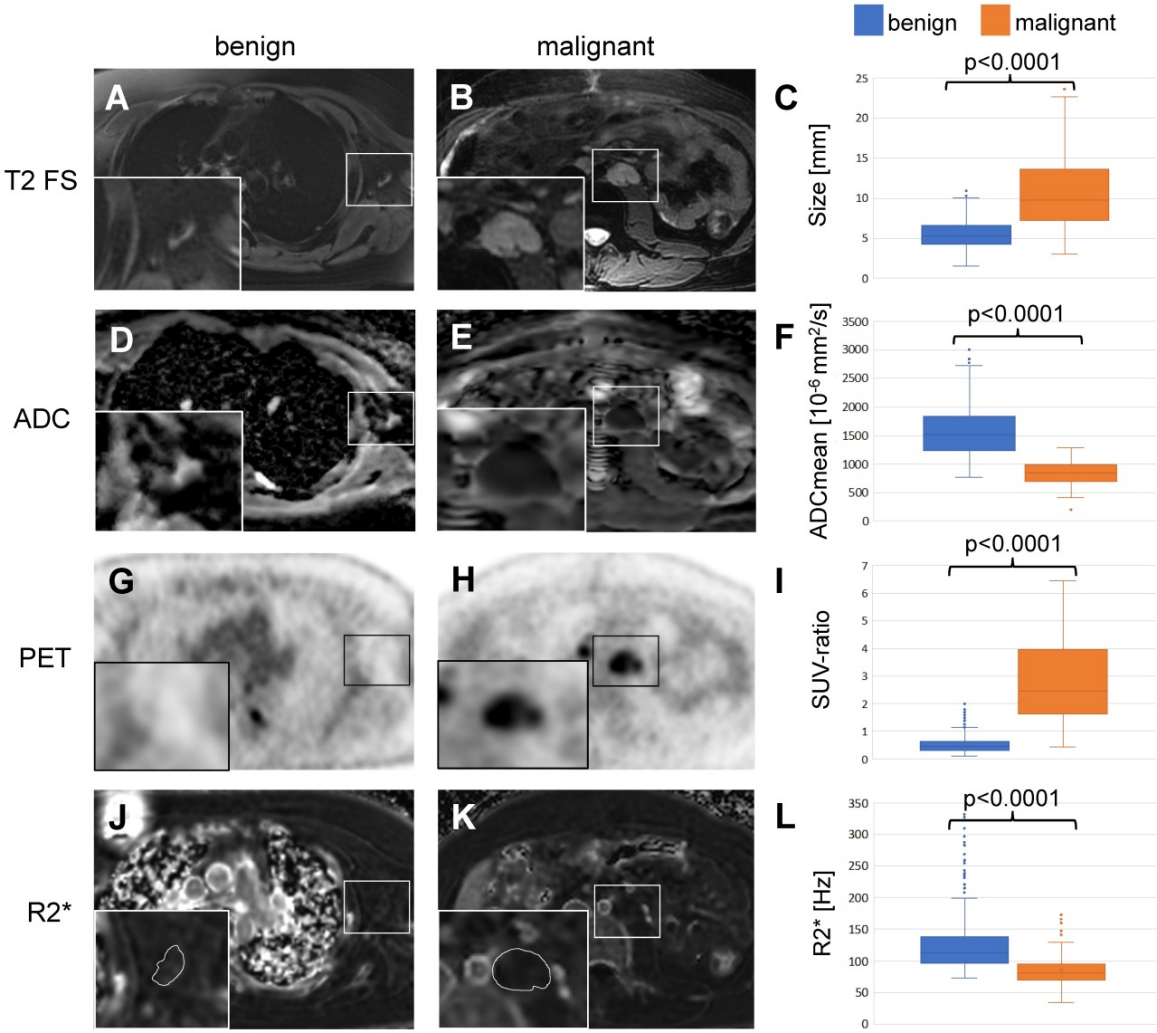
** Differentiation of benign and malignant lymph nodes in pediatric patients:** Example of a benign lymph node in the left axillary of a 16 year old boy with Ewing's sarcoma (left row) and a malignant lymph node in the left para-aortic retroperitoneal region of a 14 year old boy with a desmoplastic small round cell tumor (middle row). (A, B) Ferumoxytol-enhanced T2-weighted FSE images show a hypointense hilum of the benign lymph node (A) and lack of hypointense enhancement of the malignant node (B). (C) The mean size of 464 benign and 149 malignant lymph nodes on T2-FSE images was significantly different (p<0.0001). (D, E) Apparent diffusion coefficient (ADC) maps demonstrate unrestricted diffusion (bright signal) of the benign lymph node (D) and restricted diffusion (dark signal) of the malignant lymph node (E). (F) The mean ADC value of 464 benign and 149 malignant lymph nodes on diffusion-weighted images was significantly different (p<0.0001). (G, H) ^18^F-FDG PET images demonstrate background signal of the benign lymph node (G) and markedly increased FDG uptake of the malignant lymph node (H) compared to normal background tissue. (I) The mean standardized uptake value (SUV) ratio of 464 benign and 149 malignant lymph nodes was significantly different (p<0.0001). (J, K) R2* relaxation rate maps demonstrate increased R2* (=shortened T2*) of the benign lymph node due to increased iron content and short R2* (= long T2*) of the malignant lymph node. The mean R2* relaxation rate of 464 benign and 149 malignant lymph nodes was significantly different (p<0.0001). ^18^F-FDG: 2-deoxy-2-[F-18]fluoro-D-glucose; ADCmean: mean apparent diffusion coefficient; FSE: fast spin echo; PET: positron emission tomography; R2*: R2*-relaxation rate; SUV: standardized uptake value; SUV-ratio: SUV_max_ lymph node/SUV_mean_ liver.

There was no significant difference in quantitative data at 2 h and 24 h post ferumoxytol for ADCmean, SUV-ratio or R2* data (p=0.21, p=0.63, p=0.8, respectively; Table S4). Therefore, we included imaging studies from all time points in further analyses:

Based on the previous work from Ustabasioglu et al. an ADCmean value less than 1030 x10^-6^ mm^2^/s is considered restricted diffusion 35. Diffusion weighted images revealed restricted diffusion in 118 of 149 (79.2%) malignant lymph nodes and unrestricted diffusion in 395 of 464 (85.1%) benign lymph nodes. The ADCmean value was 1578 +/- 420 x10^-6^ mm^2^/s for benign lymph nodes and 852 +/- 206 x10^-6^ mm^2^/s for malignant lymph nodes. This difference was statistically significant (p<0.001, Figure 3D-F, Table 3).

For ^18^F-FDG PET data, the mean SUV-ratio was 0.53 +/- 0.29 for benign and 2.77 +/- 1.44 for malignant lymph nodes, which was also significantly different (p<0.001, Figure 3G-I, Table 3).

We measured the R2*-relaxation rate of entire lymph nodes, including the hilum and the parenchyma. The mean R2*-relaxation rate for benign lymph nodes was significantly higher (127.8 +/- 46.3 Hz) compared to malignant lymph nodes (84.4 +/- 23.4 Hz; p<0.001, Figure 3J-L, Table 3).

When we compared the different parameters and adjusted for size, ADCmean values were the best predictors of malignancy with an AUC of 0.99, followed closely by SUV-ratio, hilum enhancement pattern and R2* values (AUC= 0.98, 0.97, 0.93, respectively). Between ADCmean, SUV-ratio and pattern there was no significant difference (p=0.07), however all three parameters were significantly different to R2* (p=0.001, respectively; Table 2).

### Histology

**Figure 4:**
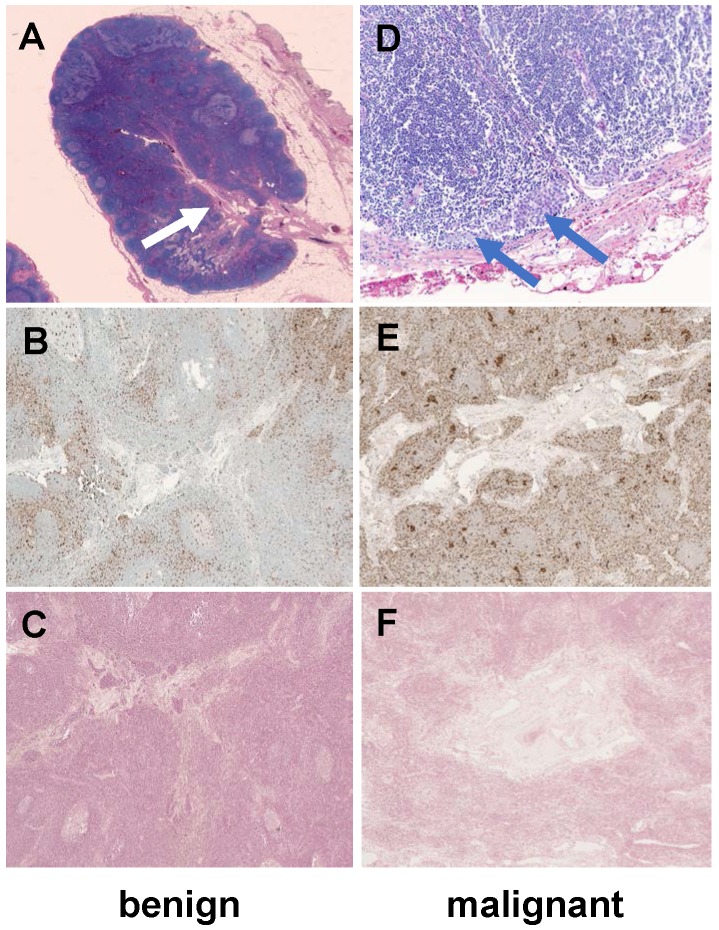
** Histology of a representative benign and malignant lymph node after ferumoxytol infusion.** The benign lymph node demonstrates a prominent central hilum with vessels (A, white arrow) on H&E staining, few scattered macrophages (brown) on CD68-stains (B, 40x magnification) and no iron in macrophages on Prussian blue staining (C, 40x magnification). The malignant lymph node demonstrates tumor cell infiltration (D, blue arrows), an increased number of macrophages on CD68-staining (E, 40x magnification) and no iron in macrophages on Prussian blue staining (F, 40x magnification). H&E: Hematoxylin and eosin.

To obtain proof-of-concept of the normal architecture of lymph nodes in a child, we obtained H&E, CD68 and Prussian blue (iron) stains of two exemplary normal lymph nodes of two of our patients: In accordance with our imaging findings, we noted a vascularized hilum and the typical morphology of medulla, paracortex and cortex (Figure 4). The cortex contained multiple lymphoid follicles. We detected tumor cell infiltrates in the malignant lymph node. We found only few, scattered macrophages in the benign lymph node. Interestingly, we found a slightly increased number of macrophages in the malignant lymph node (Figure 4). Prussian blue stains performed on sections of both nodes show no cytoplasmic iron stored within these macrophages (Figure 4), confirming our hypothesis that the iron enhancement pattern of benign lymph nodes is a function of vascularity rather than macrophage uptake.

## Discussion

Our data showed that ferumoxytol administration improves the characterization of intermediate size lymph nodes (5-10 mm) on ^18^F-FDG PET/MR imaging studies. The absence of an enhancing hilum in these subcentimeter nodes was highly suggestive of malignancy.

Our finding of a hyperintense signal of malignant lymph nodes on ferumoxytol-enhanced T2-weighted images in pediatric patients is in accordance with previous studies in adult patients 17, 29. The absent ferumoxytol-enhancement of malignant lymph nodes can be explained by lymphoid tissue replacement with malignant cells, which do not phagocytose iron oxide nanoparticles 17, 21, 36. In pediatric patients with lymphoma and markedly enlarged malignant lymph nodes, Aghighi et al. reported a significant ferumoxytol enhancement on T2-weighted MR images, which corresponded to ferumoxytol uptake by tumor associated macrophages on histology 37. It has been well described that the number of tumor associated macrophages increases with increasing tumor size 38. Absent ferumoxytol enhancement in subcentimeter lymph node metastases in our study could be either due to a lack of extravasation of nanoparticles into the lymph node interstitium or low quantity of tumor associated macrophages.

We found different ferumoxytol enhancement patterns of benign lymph nodes in children compared to those previously reported in adult patients 17. In adult patients, benign lymph nodes demonstrated hypointense signal enhancement of the entire lymph node at 24 h post injection 17, 29. These nodes demonstrated minimal or no T2*-enhancement on relatively early postcontrast scans (5 h), and progressively increasing hypointense (dark) T2* signal enhancement at 18 h and 24 h after intravenous injection of ferumoxtran-10 17 and ferumoxytol 29. Previous investigators hypothesized that the iron oxide nanoparticles were slowly phagocytosed by macrophages in normal lymph nodes 17. However, we found a different ferumoxytol enhancement pattern of benign lymph nodes in pediatric patients. We noted a marked hypointense signal enhancement of the hilum of benign lymph nodes on T2- weighted scans. This hilar enhancement did not change between 2 h and 24 h after intravenous injection of ferumoxytol. Interestingly, Wells et al. showed in benign inguinal lymph nodes in adults that after an initial increase in R2*, the pharmacokinetics in lymph nodes are similar to the distribution of ferumoxytol in the blood pool 39. It is well known from ultrasound studies 25 and histopathological investigations 22 that normal lymph nodes in children contain a highly vascularized hilum. As patients get older, the vascularity of the hilum decreases and the hilum is replaced by fat 22. Interestingly, we did not find any significant ferumoxytol enhancement of the parenchyma of benign lymph nodes in our pediatric patients, neither at 2 h nor at 24 h, suggesting that macrophage uptake does not play a role in the pediatric lymph node enhancement on MRI. This can be explained by well-described differences in cellular composition of benign lymph nodes in children and adults 22, 23, 28. In children, benign and reactive lymph nodes are better vascularized and mostly composed of T- and B-cells, with little macrophages.

Previous studies in adults show that the detection of small lymph node metastases with short axis diameter of <10 mm is a main limitation of both ^18^F-FDG PET 34, 40 and MR 34, 41 due to a limited sensitivity of PET for detection of subcentimeter lesions and absence of functional information on unenhanced MR images. Studies in animal models have shown that nanoparticles can differentiate tumor containing sentinel lymph nodes and normal lymph nodes in cervical cancer 42 and oral squamous cell cancer 43 based on differential near infrared enhancement patterns. However, both nanoparticle compounds used in these studies were not clinically applicable in humans. We found that the FDA-approved iron supplement ferumoxytol added value for the diagnosis of lymph nodes with a diameter of 5-10 mm in humans. This is important as subcentimeter malignant lymph nodes might show only mild ^18^F-FDG uptake. Positron emission tomography studies in humans revealed that tumor-targeted PET tracers increased the detection rates of small lymph node metastasis with diameters of less than 5 mm in humans with breast cancer 44 and prostate cancer 45. However, tumor-targeted tracers have not been developed for bone or soft tissue sarcomas in children. Our approach to visualize the hilum with ferumoxytol was not useful for normal lymph nodes with a size of < 5 mm, likely due to limitations in anatomical resolution. However, our data indicate that the likelihood of malignancy in such small lymph nodes is extremely low and micrometastasis would not change the treatment or outcome of patients. Currently the standard of evaluating and detecting micrometastatic disease burden is through polymerase chain reaction and flow cytometry as it can detect as few as 1 tumor cell in 500 000 cells 46 which is more sensitive than any other clinical imaging technique. Yet, the presence of micrometastases in children with neuroblastoma 47 or Ewing sarcoma 48 did not affect the initial disease burden, event-free or overall survival in patients. Therefore, detection of micrometastases does not alter patient management. In patients with bone sarcomas, pulmonary metastases smaller than 3 mm are not referred to surgery because they are too small to be localized, cannot be reliably resected and because the minimal disease burden will likely not change overall outcome 49, 50.

Due to recent concerns about gadolinium deposition in the brain, there is a search for alternative MR contrast agents that are biodegradable. Iron oxide nanoparticles could fill this gap. The off-label use of iron supplements such as ferumoxytol as MR contrast agents is an example of a therapeutic drug that can be used for diagnostic purposes. Future studies could investigate, if the hilar accumulation of nanoparticles can facilitate priming of antigen presenting dendritic immune cells and enhance the anti-tumor effect of standard chemotherapy. Other studies showed that the tumor environment releases immunosuppressive cytokines 51. Presentation of whole-tumor cell lysate vaccines caused limited activation of the immune system against the cancer 52. Kohlhapp et al. showed that coupling tumor antigens to nanoparticles activated T-cells and reduced tumor growth 53. Other groups showed that nanoparticles coupled with cancer antigen delayed tumor growth and prolonged survival 54. Nanoparticles with a relatively small size of 25 nm induced antigen presentation and dendritic cell activation in lymph nodes 55.

Our results showed that ADCmean values, SUV-ratio, and hilar enhancement pattern had similar high prediction rates for lymph node malignancy. Several studies described the high diagnostic value of ADC maps in pediatric patients 56, 57. Ferumoxytol-enhancement pattern and ADCmean yielded the same predictive information than SUV-values on PET, yet without any radiation exposure. This is very important because ionizing radiation exposure can be avoided and MR scanners are more widely available than PET scanners.

In our study, a few limitations should be noted: 1. Participants in our study had a variety of different tumor types. However, our sample is representative of pediatric patients typically investigated in a major Children's Hospital, especially since lymph node metastases are very rare in pediatric cancers. 2. Our study evaluated patients before start of chemotherapy or irradiation. Further studies have to evaluate ferumoxytol-enhancement patterns of benign and malignant lymph nodes after therapy. 3. As ferumoxytol has to be injected slowly over 15 min we did not acquire pre-contrast scans in all patients. To acquire two separate PET/MRI scans without and with ferumoxytol would not be feasible for routine clinical applications in children especially given the radiation dose associated with a PET scan.

## Conclusions

In summary, we found that MR enhancement pattern of lymph nodes with ferumoxytol are different in children compared to adults. For lymph nodes with a size of 5-10 mm, the presence of an enhancing hilum can reassure the diagnosis of a benign lymph node.

## Supplementary Material

Supplementary figures and tables.

## Figures and Tables

**Table 1:** Distribution of pattern in benign and malignant lymph nodes

	Hilum ↓ Parenchyma ↑ 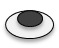			Hilum ↑ Parenchyma ↑ 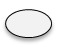	
Sizes	benign	malignant		benign	malignant
< 5 mm	112	0		74	9
≥ 5 - < 10 mm	240	5		27	64
≥ 10 mm	10	0		1	71
**All**	**362**	**5**		**102**	**144**

**Table 2:** Predictive value of quantitative parameters

Parameter	Odds ratio	95% Confidence interval		p-value	AUC
		lower	upper		
ADCmean	0.99	0.99	0.99	**<0.001**	0.989 (0.984-0.995)
SUV-ratio	10.82	4.18	28.03	**<0.001**	0.979 (0.967-0.991)
Pattern	10.70	3.94	29.11	**<0.001**	0.974 (0.961-0.987)
R2*	1.31	1.22	1.40	**<0.001**	0.933 (0.906-0.961)

ADCmean: Mean apparent diffusion coefficient; SUV: standardized uptake value; SUV-ratio: SUVmax lesion/SUVmean liver; R2*: R2*-relaxation rate; AUC: area under the curve

**Table 3:** Differentiation of benign and malignant lymph nodes in pediatric patients on ferumoxytol-enhanced ^18^F- FDG-PET/MRI scans

Sizes	ADCmean [10^-6^ mm^2^/s]		SUV-ratio		R2* [Hz]	
	benign	malignant	benign	malignant	benign	malignant
< 5 mm	1521.0 ± 416.6	851.7 ± 121.0	0.5 ± 0.2	1.2 ± 0.5	123.5 ± 45.9	92.3 ± 23.7
≥ 5 - < 10 mm	1614.1 ± 418.9	818.2 ± 188.1	0.57 ± 0.3	2.3 ± 1.2	130.9 ± 47.9	91.7 ± 27.9
≥ 10 mm	1539.3 ± 432.7	884.2 ± 227.4	0.7 ± 0.3	3.4 ± 1.4	128.3 ± 32.5	76.7 ± 17.9
**All**	**1578.7 ± 419.8**	**851.7 ± 206.2**	**0.5 ± 0.3**	**2.8 ± 1.5**	**127.8 ± 46.3**	**84.4 ± 23.4**

Mean +/- standard deviation; ADCmean: Mean apparent diffusion coefficient; SUV: standardized uptake value; SUV-ratio: SUVmax lesion/SUVmean liver; R2*: R2*-relaxation rate

## Abbreviations

^18^F-FDG: 2-deoxy-2-[F-18]fluoro-D-glucose
ADC: apparent diffusion coefficient
AUC: area under the curve
CI: confidence interval
CT: computed tomography
DWI: diffusion weighted imaging
FOV: field of view
IDEAL-IQ: iterative decomposition of water and fat with echo asymmetry and least-squares estimation
IND: investigational new drug
LAVA: liver acquisition with volume acquisition
MRI: magnetic resonance imaging
OR: odds ratio
PET: positron emission tomography
PROPELLER: periodically rotated overlapping parallel lines with enhanced reconstruction
R2*: R2*-relaxation rate
RECIST: response evaluation criteria for solid tumors
ROC: receiver operating curve
SUV: standardized uptake value
SUV-ratio: SUVmax lesion/SUVmean liver
T2-FSE: T2-weighted fast spin echo

## References

[1] Rodeberg DA, Garcia-Henriquez N, Lyden ER, Davicioni E, Parham DM, Skapek SX (2011). Prognostic significance and tumor biology of regional lymph node disease in patients with rhabdomyosarcoma: a report from the Children's Oncology Group. J Clin Oncol.

[2] Sawamura C, Matsumoto S, Shimoji T, Ae K, Okawa A (2013). Lymphadenectomy and histologic subtype affect overall survival of soft tissue sarcoma patients with nodal metastases. Clin Orthop Relat Res.

[3] Toth GB, Varallyay CG, Horvath A, Bashir MR, Choyke PL, Daldrup-Link HE (2017). Current and potential imaging applications of ferumoxytol for magnetic resonance imaging. Kidney Int.

[4] Na DG, Lim HK, Byun HS, Kim HD, Ko YH, Baek JH (1997). Differential diagnosis of cervical lymphadenopathy: usefulness of color Doppler sonography. AJR Am J Roentgenol.

[5] Ishiguchi H, Ito S, Kato K, Sakurai Y, Kawai H, Fujita N (2018). Diagnostic performance of (18)F-FDG PET/CT and whole-body diffusion-weighted imaging with background body suppression (DWIBS) in detection of lymph node and bone metastases from pediatric neuroblastoma. Ann Nucl Med.

[6] Dorfman RE, Alpern MB, Gross BH, Sandler MA (1991). Upper abdominal lymph nodes: criteria for normal size determined with CT. Radiology.

[7] Dooms GC, Hricak H, Crooks LE, Higgins CB (1984). Magnetic resonance imaging of the lymph nodes: comparison with CT. Radiology.

[8] Cheson BD, Fisher RI, Barrington SF, Cavalli F, Schwartz LH, Zucca E (2014). Recommendations for initial evaluation, staging, and response assessment of Hodgkin and non-Hodgkin lymphoma: the Lugano classification. J Clin Oncol.

[9] Federico SM, Spunt SL, Krasin MJ, Billup CA, Wu J, Shulkin B (2013). Comparison of PET-CT and conventional imaging in staging pediatric rhabdomyosarcoma. Pediatr Blood Cancer.

[10] Hurley C, McCarville MB, Shulkin BL, Mao S, Wu J, Navid F (2016). Comparison of (18) F-FDG-PET-CT and Bone Scintigraphy for Evaluation of Osseous Metastases in Newly Diagnosed and Recurrent Osteosarcoma. Pediatr Blood Cancer.

[11] Uslu L, Donig J, Link M, Rosenberg J, Quon A, Daldrup-Link HE (2015). Value of 18F-FDG PET and PET/CT for evaluation of pediatric malignancies. J Nucl Med.

[12] Knight PJ, Mulne AF, Vassy LE (1982). When is lymph node biopsy indicated in children with enlarged peripheral nodes?. Pediatrics.

[13] Ulaner GA, Lilienstein J, Gonen M, Maragulia J, Moskowitz CH, Zelenetz AD (2014). False-Positive [18F]fluorodeoxyglucose-avid lymph nodes on positron emission tomography-computed tomography after allogeneic but not autologous stem-cell transplantation in patients with lymphoma. J Clin Oncol.

[14] Malik AI, Akhtar N, Loya A, Yusuf MA (2014). Endoscopic ultrasound - fine needle aspiration of 2-deoxy-2-[18F] fluoro-D-glucose avid lymph nodes seen on positron emission tomography- computed tomography -what looks like cancer may not always be so. Cancer Imaging.

[15] Schaefer NG, Taverna C, Strobel K, Wastl C, Kurrer M, Hany TF (2007). Hodgkin disease: diagnostic value of FDG PET/CT after first-line therapy-is biopsy of FDG-avid lesions still needed?. Radiology.

[16] Connolly AA, MacKenzie K (1997). Paediatric neck masses-a diagnostic dilemma. J Laryngol Otol.

[17] Harisinghani MG, Barentsz J, Hahn PF, Deserno WM, Tabatabaei S, van de Kaa CH (2003). Noninvasive detection of clinically occult lymph-node metastases in prostate cancer. N Engl J Med.

[18] Thoeny HC, Triantafyllou M, Birkhaeuser FD, Froehlich JM, Tshering DW, Binser T (2009). Combined ultrasmall superparamagnetic particles of iron oxide-enhanced and diffusion-weighted magnetic resonance imaging reliably detect pelvic lymph node metastases in normal-sized nodes of bladder and prostate cancer patients. Eur Urol.

[19] Birkhauser FD, Studer UE, Froehlich JM, Triantafyllou M, Bains LJ, Petralia G (2013). Combined ultrasmall superparamagnetic particles of iron oxide-enhanced and diffusion-weighted magnetic resonance imaging facilitates detection of metastases in normal-sized pelvic lymph nodes of patients with bladder and prostate cancer. Eur Urol.

[20] Corot C, Robert P, Idee JM, Port M (2006). Recent advances in iron oxide nanocrystal technology for medical imaging. Adv Drug Deliv Rev.

[21] Heesakkers RA, Hovels AM, Jager GJ, van den Bosch HC, Witjes JA, Raat HP (2008). MRI with a lymph-node-specific contrast agent as an alternative to CT scan and lymph-node dissection in patients with prostate cancer: a prospective multicohort study. Lancet Oncol.

[22] Luscieti P, Hubschmid T, Cottier H, Hess MW, Sobin LH (1980). Human lymph node morphology as a function of age and site. J Clin Pathol.

[23] Ramsay AD (2004). Reactive lymph nodes in pediatric practice. Am J Clin Pathol.

[24] Hadamitzky C, Spohr H, Debertin AS, Guddat S, Tsokos M, Pabst R (2010). Age-dependent histoarchitectural changes in human lymph nodes: an underestimated process with clinical relevance?. J Anat.

[25] Turgut E, Celenk C, Tanrivermis Sayit A, Bekci T, Gunbey HP, Aslan K (2017). Efficiency of B-mode Ultrasound and Strain Elastography in Differentiating Between Benign and Malignant Cervical Lymph Nodes. Ultrasound Q.

[26] Gruver AL, Hudson LL, Sempowski GD (2007). Immunosenescence of ageing. J Pathol.

[27] Lazuardi L, Jenewein B, Wolf AM, Pfister G, Tzankov A, Grubeck-Loebenstein B (2005). Age-related loss of naive T cells and dysregulation of T-cell/B-cell interactions in human lymph nodes. Immunology.

[28] Gomez CR, Boehmer ED, Kovacs EJ (2005). The aging innate immune system. Curr Opin Immunol.

[29] Harisinghani M, Ross RW, Guimaraes AR, Weissleder R (2007). Utility of a new bolus-injectable nanoparticle for clinical cancer staging. Neoplasia.

[30] Mattsson S, Johansson L, Leide Svegborn S, Liniecki J, Nosske D, Riklund KA (2015). Radiation Dose to Patients from Radiopharmaceuticals: a Compendium of Current Information Related to Frequently Used Substances. Ann ICRP.

[31] van Persijn van Meerten EL, Gelderblom H, Bloem JL (2010). RECIST revised: implications for the radiologist. A review article on the modified RECIST guideline. Eur Radiol.

[32] Eisenhauer EA, Therasse P, Bogaerts J, Schwartz LH, Sargent D, Ford R (2009). New response evaluation criteria in solid tumours: revised RECIST guideline (version 1.1). Eur J Cancer.

[33] Wood JC, Enriquez C, Ghugre N, Tyzka JM, Carson S, Nelson MD (2005). MRI R2 and R2* mapping accurately estimates hepatic iron concentration in transfusion-dependent thalassemia and sickle cell disease patients. Blood.

[34] Ganeshalingam S, Koh DM (2009). Nodal staging. Cancer Imaging.

[35] Ustabasioglu FE, Samanci C, Alis D, Samanci NS, Kula O, Olgun DC (2017). Apparent Diffusion Coefficient Measurement in Mediastinal Lymphadenopathies: Differentiation between Benign and Malignant Lesions. J Clin Imaging Sci.

[36] Harisinghani MG, Dixon WT, Saksena MA, Brachtel E, Blezek DJ, Dhawale PJ (2004). MR lymphangiography: imaging strategies to optimize the imaging of lymph nodes with ferumoxtran-10. Radiographics.

[37] Aghighi M, Theruvath AJ, Pareek A, Pisani LL, Alford R, Muehe AM (2018). Magnetic Resonance Imaging of Tumor-Associated Macrophages: Clinical Translation. Clin Cancer Res.

[38] Ruffell B, Affara NI, Coussens LM (2012). Differential macrophage programming in the tumor microenvironment. Trends Immunol.

[39] Wells SA, Schubert T, Motosugi U, Sharma SD, Campo CA, Kinner S (2020). Pharmacokinetics of Ferumoxytol in the Abdomen and Pelvis: A Dosing Study with 1.5- and 3.0-T MRI Relaxometry. Radiology.

[40] Kim BT, Lee KS, Shim SS, Choi JY, Kwon OJ, Kim H (2006). Stage T1 non-small cell lung cancer: preoperative mediastinal nodal staging with integrated FDG PET/CT-a prospective study. Radiology.

[41] Jager GJ, Barentsz JO, Oosterhof GO, Witjes JA, Ruijs SJ (1996). Pelvic adenopathy in prostatic and urinary bladder carcinoma: MR imaging with a three-dimensional TI-weighted magnetization-prepared-rapid gradient-echo sequence. AJR Am J Roentgenol.

[42] Wei R, Jiang G, Lv M, Tan S, Wang X, Zhou Y (2019). TMTP1-modified Indocyanine Green-loaded Polymeric Micelles for Targeted Imaging of Cervical Cancer and Metastasis Sentinel Lymph Node in vivo. Theranostics.

[43] Wang Y, Zhang W, Sun P, Cai Y, Xu W, Fan Q (2019). A Novel Multimodal NIR-II Nanoprobe for the Detection of Metastatic Lymph Nodes and Targeting Chemo-Photothermal Therapy in Oral Squamous Cell Carcinoma. Theranostics.

[44] Zhang J, Mao F, Niu G, Peng L, Lang L, Li F (2018). (68)Ga-BBN-RGD PET/CT for GRPR and Integrin alphavbeta3 Imaging in Patients with Breast Cancer. Theranostics.

[45] Jilg CA, Drendel V, Rischke HC, Beck T, Vach W, Schaal K (2017). Diagnostic Accuracy of Ga-68-HBED-CC-PSMA-Ligand-PET/CT before Salvage Lymph Node Dissection for Recurrent Prostate Cancer. Theranostics.

[46] Dubois SG, Epling CL, Teague J, Matthay KK, Sinclair E (2010). Flow cytometric detection of Ewing sarcoma cells in peripheral blood and bone marrow. Pediatr Blood Cancer.

[47] Burchill SA (2004). Micrometastases in neuroblastoma: are they clinically important?. J Clin Pathol.

[48] Vo KT, Edwards JV, Epling CL, Sinclair E, Hawkins DS, Grier HE (2016). Impact of Two Measures of Micrometastatic Disease on Clinical Outcomes in Patients with Newly Diagnosed Ewing Sarcoma: A Report from the Children's Oncology Group. Clin Cancer Res.

[49] Cipriano C, Brockman L, Romancik J, Hartemayer R, Ording J, Ginder C (2015). The Clinical Significance of Initial Pulmonary Micronodules in Young Sarcoma Patients. J Pediatr Hematol Oncol.

[50] Absalon MJ, McCarville MB, Liu T, Santana VM, Daw NC, Navid F (2008). Pulmonary nodules discovered during the initial evaluation of pediatric patients with bone and soft-tissue sarcoma. Pediatr Blood Cancer.

[51] Wu AA, Drake V, Huang HS, Chiu S, Zheng L (2015). Reprogramming the tumor microenvironment: tumor-induced immunosuppressive factors paralyze T cells. Oncoimmunology.

[52] Chiang CL, Kandalaft LE, Coukos G (2011). Adjuvants for enhancing the immunogenicity of whole tumor cell vaccines. Int Rev Immunol.

[53] Kohlhapp F, Huelsmann E, Rudra J, Nabatiyan A, Zloza A (2015). Single-step nanoparticle antigen presentation system for tumor immunotherapy.

[54] de Faria PC, dos Santos LI, Coelho JP, Ribeiro HB, Pimenta MA, Ladeira LO (2014). Oxidized multiwalled carbon nanotubes as antigen delivery system to promote superior CD8(+) T cell response and protection against cancer. Nano Lett.

[55] Reddy ST, van der Vlies AJ, Simeoni E, Angeli V, Randolph GJ, O'Neil CP (2007). Exploiting lymphatic transport and complement activation in nanoparticle vaccines. Nat Biotechnol.

[56] Klenk C, Gawande R, Uslu L, Khurana A, Qiu D, Quon A (2014). Ionising radiation-free whole-body MRI versus (18)F-fluorodeoxyglucose PET/CT scans for children and young adults with cancer: a prospective, non-randomised, single-centre study. Lancet Oncol.

[57] Gawande RS, Gonzalez G, Messing S, Khurana A, Daldrup-Link HE (2013). Role of diffusion-weighted imaging in differentiating benign and malignant pediatric abdominal tumors. Pediatr Radiol.

