# Supplementary material for: Differentiation of benign and malignant lymph nodes in pediatric patients on ferumoxytol-enhanced PET/MRI
Source: Theranostics. 2020 Feb 18;10(8):3612–21. doi: 10.7150/thno.40606 (PMC7069081; doi:10.7150/thno.40606)
Supplement: Supplementary file 1 — Supplementary figures and tables. [file thnov10p3612s1.pdf]

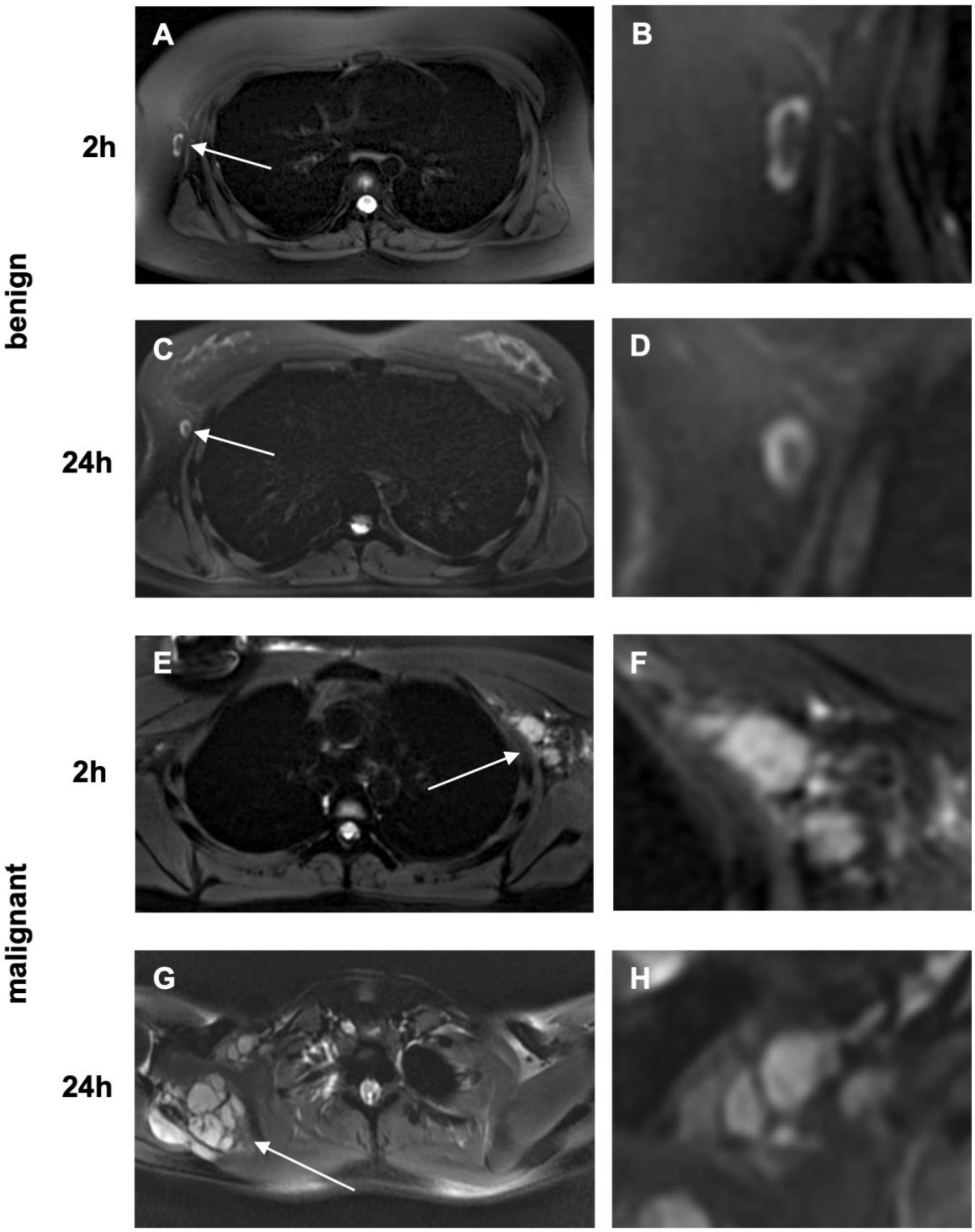

Figure S1: Morphology of lymph nodes at 2 h and 24 h after ferumoxytol infusion. Axial T2 PROPELLER images show morphology of lymph nodes at (A) 2h after ferumoxytol infusion (B) shows benign node with hypointense hilum and hyperintense cortex. The same pattern is visible at (C) 24h after ferumoxytol infusion in (D) benign lymph nodes. In (E) malignant lymph nodes at 2h (F) a homogenous hyperintense pattern is displayed. This also applies for (G) 24h after ferumoxytol infusion (H) no hilum is discernible.

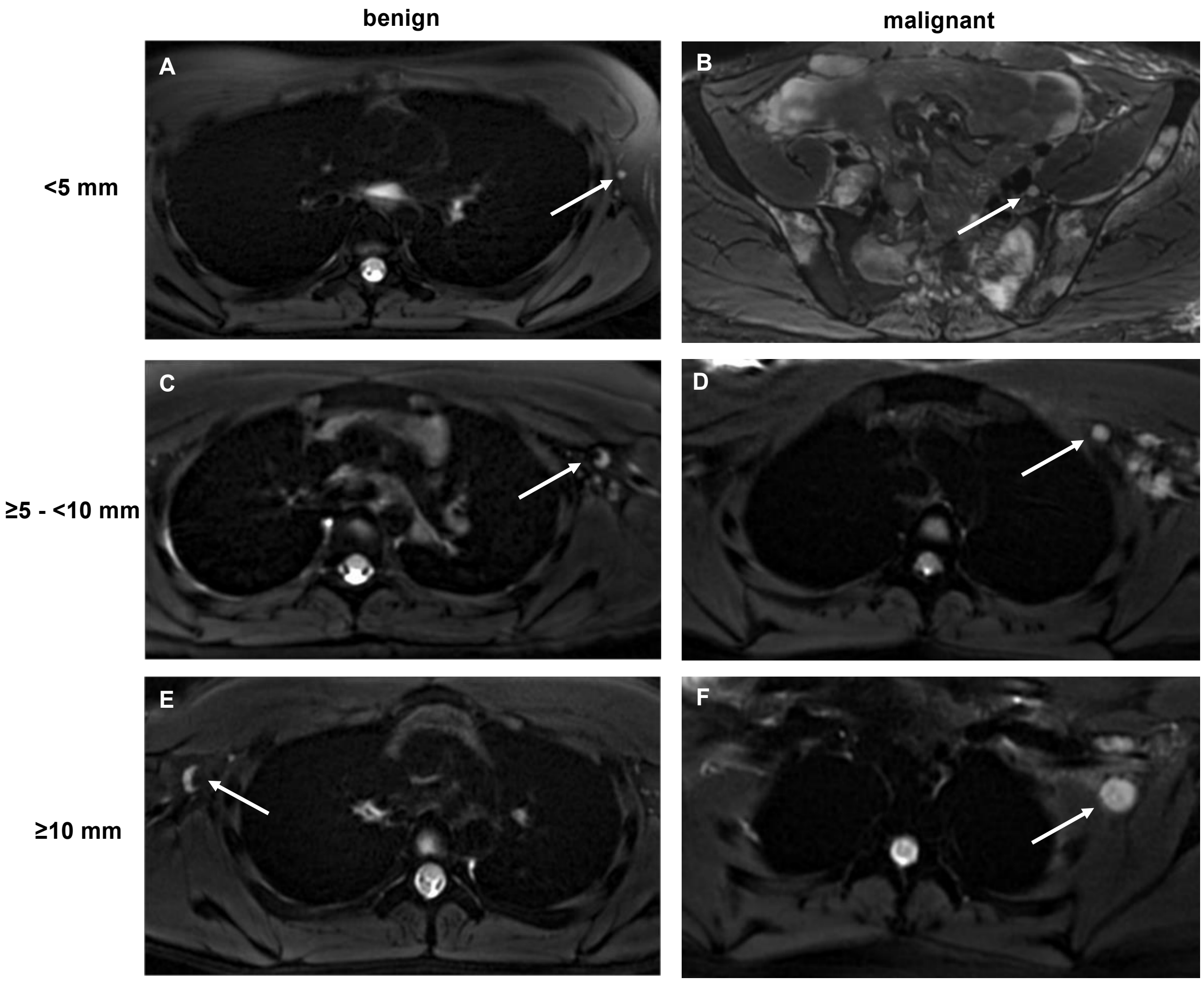

Figure S2: Morphologies of different sizes of benign and malignant lymph nodes after ferumoxytol infusion: Axial ferumoxytol-enhanced T2-FSE and PROPELLER images of benign lymph nodes with a short axis of (A) <5 mm often show no T2-hypointense hilum, it is easily visible in lymph nodes (C) with a size between 5-10 mm and (E) ≥10 mm. In malignant lymph nodes with a short axis of (B) <5 mm, (D) ≥5 - <10 mm and (F) ≥10 mm a homogenously T2-hyperintense pattern without a recognizable ferumoxytol-enhancement is present. The specific enhancement-pattern especially helps depict benign and malignant lymph nodes in the range between ≥5 - <10 mm where size has a bad prediction of malignancy.

T2-FSE: T2-weighted fast spin echo.

Table S1: Patient demographics

Patient ID	Age (years)	Sex	Diagnosis	Hours post ferumoxytol infusion
1	16	F	Nasopharyngeal carcinoma	24
2	16	F	Ovarian steroid cell tumor	24
3	11	M	Wilms Tumor	24
4	15	F	Ewing sarcoma	24
5	13	M	Ewing sarcoma	24
6	15	M	Hepatocellular carcinoma	24
7	18	M	Osteosarcoma	24
8	13	F	Osteosarcoma	24
9	16	M	Lipoma	2
10	17	M	Gastrointestinal stromal tumor	24
11	14	M	Osteosarcoma	2
12	10	M	Synovial sarcoma	2
13	14	M	Desmoplastic small round cell tumor	2
14	10	M	Undifferentiated sarcoma	2
15	8	M	Malignant peripheral nerve sheath tumor	2
16	8	M	Synovial sarcoma	2
17	14	M	Osteosarcoma	2
18	11	M	Rhabdomyosarcoma	24
19	16	M	Ewing sarcoma	24
20	11	M	Langerhans cell histiocytosis	24
21	8	F	Rhabdomyosarcoma	24
22	16	M	Rhabdomyosarcoma	24
23	15	M	Ewing sarcoma	24
24	18	M	Osteosarcoma	24
25	17	F	Osteosarcoma	24
26	14	M	Osteosarcoma	24
27	7	F	Synovial sarcoma	2
28	9	M	Osteosarcoma	2
29	11	F	Langerhans cell histiocytosis	24
30	14	M	Undifferentiated sarcoma	24
31	11	F	Osteosarcoma	2
32	14	F	Germ cell tumor	24
33	18	F	Wilms Tumor	2
34	11	M	Langerhans cell histiocytosis	2
35	15	F	Langerhans cell histiocytosis	2
36	14	M	Langerhans cell histiocytosis	2
37	15	M	Langerhans cell histiocytosis	2
38	11	M	Osteosarcoma	2
39	13	F	Osteosarcoma	2
40	16	M	Desmoplastic small round cell tumor	2
41	18	M	Desmoplastic small round cell tumor	2
42	18	M	Neuroblastoma	24

M = male; F = female

Table S2: MRI and PET parameter

Parameter	axial T2w FS	axial T2w FS	axial DWI	axial IDEAL-IQ	PET
Sequence type	FSE	PROPELLER	EPI	DIXON	N/A
TE [ms]	103	112	59	6 echoes (1.2-6.5)	N/A
TR [ms]	8331	6510	4457	6.2	N/A
Matrix size [pixel]	384 x 320	384 x 384	128 x 72	160 x 160	-
FOV [cm]	48	48	48	48	25
Slice thickness [mm]	4	4	6	6	2.8
b-value [s/mm^2]	-	-	50, 600	-	-
FA [°]	111	110	90	5	-
Scan duration [min]	3:15	6:20	01:21	00:24	3:30

DWI: diffusion weighted imaging; EPI: echo planar imaging; IDEAL-IQ: iterative decomposition of water and fat with echo asymmetry and least-squares estimation; FA: flip angle; FOV: field of view; FS: fat saturated; FSE: fast spin echo; PET: positron emission tomography; PROPELLER: periodically rotated overlapping parallel lines with enhanced reconstruction; T2w: T2-weighted; TE: echo time; TR: repetition time.

Table S3: Morphology of all lymph nodes according to size and time point of imaging after ferumoxytol infusion

	Morphology	Benign				Malignant				All
		<5 mm	≥5 - <10 mm	≥10 mm	∑	<5 mm	≥5 - <10 mm	≥10 mm	∑	∑
**post 24 h**	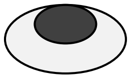	84	127	4	215	0	2	0	2	**217**
	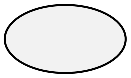	35	6	1	42	7	23	16	46	**88**
	**Total lymph nodes**	119	133	5	257	7	25	16	48	**305**
**post 2 h**	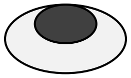	28	113	6	147	0	3	0	3	**150**
	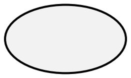	39	21	0	60	2	41	55	98	**158**
	**Total lymph nodes**	67	134	6	207	2	44	55	101	**308**
**all LN (2 + 24 h)**	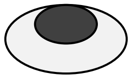	112	240	10	362	0	5	0	5	**367**
	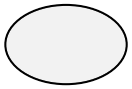	74	27	1	102	9	64	71	144	**246**
	**Total lymph nodes**	**186**	**267**	**11**	**464**	**9**	**69**	**71**	**149**	**613**

Table S4: Quantitative data of benign and malignant lymph nodes at 2 h vs 24 h

	**ADCmean [10^{-6} mm^2/s]**		**SUV-ratio**		**R2* [Hz]**	
	benign	malignant	benign	malignant	benign	malignant
2 h	1616.9 ± 407.4	869.1 ± 179.4	0.49 ± 0.24	2.65 ± 1.08	131.6 ± 49.8	85.2 ± 25.1
24 h	1554.3 ± 427.3	754.4 ± 164.1	0.55 ± 0.32	1.48 ± 0.94	124.6 ± 42.9	82.6 ± 23.0
All	1578.7 ± 419.8	851.7 ± 206.2	0.53 ± 0.29	2.77 ± 1.45	127.8 ± 46.3	84.4 ± 23.4

Mean +/- standard deviation. ADCmean: mean apparent diffusion coefficient; SUV: standardized uptake value; SUV-ratio: SUVmax lesion/SUVmean liver; R2: R2*-relaxation rate.*
